# Reading body and face language in male schizophrenia

**DOI:** 10.1038/s41537-026-00775-6

**Published:** 2026-07-07

**Authors:** Annika Resch, Alexander N. Sokolov, Patrick Steinwand, Andreas J. Fallgatter, Marina A. Pavlova

**Affiliations:** 1 Department of Psychiatry and Psychotherapy, Medical School and University Hospital, Eberhard Karls University of Tübingen, Tübingen, Germany; 2 German Center for Mental Health (DZPG), partner site Tübingen, Tübingen, Germany

**Keywords:** Schizophrenia, Emotion

## Abstract

Neurotypical people are generally quite adept at interpreting social signals from dynamic bodies and faces. This ability prevents one from incurring high costs associated with ineffective and maladaptive social interactions. Individuals with mental disorders, such as schizophrenia (SZ), often exhibit deficits in nonverbal social cognition. It remains unclear whether reading the language of bodies and faces is affected by SZ, and if this is indeed the case, how these potential deficits are related to one another. In the present study, participants (28 males with SZ and 28 typically developing, TD, matched controls) were administered face-to-face computer tasks on inferring emotions from dynamic point-light body motion and faces. The outcome indicates that SZ patients exhibit global impairments in both body and face reading, albeit patients demonstrate a similar emotion recognition profile as TD controls. In SZ patients only, a positive link was found between accuracy of recognizing emotions expressed through faces and bodies, whereas processing speed of emotions conveyed through bodies and faces was tied to each other in both SZ and TD individuals. For SZ patients, inferring social signals from dynamic faces and bodies may be rather challenging in terms of neurocognitive mechanisms, which is reflected in the tight link in recognition accuracy. Along with previous data on inferring emotions in the eyes collected in the same cohort, this work provides novel insights into the specific global aberrations in social cognition in SZ and offers a blueprint for the development of strategies for the targeted treatment of gender-specific mental disorders.

## Introduction

Social cognition represents a core function that is crucial for effective management of daily interactions. The integration of signals from the body with facial information allows the comprehension of others’ mental and emotional states, which prevents paying high costs for ineffective social interaction. However, adaptive social interaction may be rather challenging for people with mental disorders^1–8^.

It appears that distinct mental disorders exhibit disorder-specific profiles of social cognition deficits. For example, individuals with major depressive disorder (MDD) demonstrate selectivity in recognition of facial affect, with the most severe impairments in inferring sadness^9–11^, in perception of social interaction in point-light body motion^12^, as well as in inferring happiness^13^ and neutral expressions^14^ through dynamic body language. People with bipolar disorder also exhibit selective impairments in emotion recognition, with deficits primarily affecting facial emotion processing, particularly sadness recognition, and a bias toward interpreting neutral faces as sad, while emotion recognition from body motion remains largely intact^15^.

By contrast, schizophrenia (SZ) is thought to be characterized by a global impairment in social cognition, affecting multiple facets of nonverbal communication^16,17^. These deficits may substantially influence the ability of individuals with SZ to form and maintain social relationships, resulting in social avoidance and loneliness^18–21^. Patients with SZ not only experience difficulties in reading the language of the eyes as assessed by the Reading the Mind in the Eyes Test (RMET)^1,16,17,22–25^, but also in inferring social signals from faces^26,27^ and dynamic bodies^28–34^; for review, see refs. ^31,35,36^.

Point-light displays portraying a human figure by a set of light dots on the head and main joints of an otherwise invisible body^37^ provide a proper tool for investigation of body language reading as participants can rely solely on the motion and are not influenced by other cues such as gender or body shape^38^. Neurotypical people readily recognize emotions represented by moving point-light bodies^39–46^, whereas individuals with SZ and even individuals with high-risk of psychosis^47^ show deficient recognition of basic emotions through body motion^29,30,32^. SZ has recently been demonstrated to be associated with a more general profile of deficits, including difficulties in interpreting social interactions in dyads of point-light actors^34^.

Along with difficulties in body language reading, alterations in the processing of facial information have been found in SZ. Patients with SZ exhibit lower sensitivity to a coarse face scheme in images eliciting face pareidolia (seeing faces in non-face images such as shadows)^4,48,49^ as well as in facial affect recognition^50–52^. In daily life, we deal mostly with dynamic faces^45,53^. Similar to point-light body motion, point-light faces consist of a number of white dots placed on black-colored faces expressing various emotions^54^. In TD individuals, point-light faces are reported to provide sufficient information not only to identify them as faces, but also to reliably recognize facial affect^55–58^. All these findings raise the question of whether and, if so, how alterations in the interpretation of emotional expressions of the body and face are interconnected in SZ.

Males are affected by SZ 1.4–1.6 times as often as females^59,60^ and differ from them not only in clinical manifestation, such as age of illness onset, symptom profiles, and severity, but also in underlying brain structure and function^59–62^. In particular, males with SZ typically show an earlier onset of the disorder and a more severe clinical presentation, including poorer premorbid functioning and more severe negative symptoms such as social withdrawal, blunted or incongruent affect^63–65^.

The findings on gender differences in social cognition in SZ remain inconclusive^63,66^. While some studies report a disadvantage for males compared to females^67–72^, others fail to find gender differences, at least for some tasks^73–75^. Ultimately, gender should be taken into account when studying social cognition. To minimize possible gender-related variability, the present study focused on male patients with SZ.

In this piece of research, we examined: (i) whether the capabilities of inferring emotions through dynamic point-light faces and bodies are aberrant in male SZ as compared with TD individuals; (ii) if so, whether this deficit is selective, i.e., specific only for some tasks or global; and (iii) whether the dis/abilities for inferring emotions from faces and bodies are interrelated in SZ in terms of accuracy and processing speed.

## Methods

### Participants

Fifty-six participants were enrolled in the study. The cohort of SZ patients was recruited from inpatient units at the Department of Psychiatry and Psychotherapy, University Hospital Tübingen, Germany. Patients were aged 31.54 ± 10.57 years (mean ± standard deviation, SD; median, Mdn, 28 years, 95% confidence interval, CI [27.44; 35.64]; age range, 18–54 years). Eighteen out of 28 patients were diagnosed with paranoid SZ (International Statistical Classification of Diseases and Related Health Problems, 10^th^ Revision, ICD-10; F20.0), one patient with a special form of SZ (cenesthesia; F20.8), one with schizotypal disorder (F21), and eight patients with schizoaffective disorder [two of them of manic type (F25.0), two of depressive type (F25.1), and four of mixed type (F25.2)]. The average time from the first diagnosis till examination was 7.12 ± 9.66 years (Mdn, 4 years; 95% CI [3.13; 11.11]). Seventeen out of 28 SZ patients had one or more comorbidities such as abuse of nicotine, alcohol, and other drugs (Table S1, Supplementary Material). Twenty-seven out of 28 patients were under medication (Supplementary Material). The data of the same sample of SZ patients and TD controls on the RMET and emotion recognition in masked faces (EMF) are reported in earlier work^17^.

A total of 28 TD individuals, who were recruited from the local community, were matched to the patients in terms of gender and age (31.64 ± 11.1 years; Mdn, 28 years, 95% CI [27.34; 35.94]; age range, 21–57 years). No significant difference in age was observed between SZ patients and TD controls (Mann–Whitney test, *U* = 386, *p* = 0.928, two-tailed, n.s.). None had a history of neurological or mental disorders (including SZ, major depressive disorder (MDD), autism spectrum disorders (ASD), and attention deficit hyperactivity disorder (ADHD)) or regular medication intake.

All subjects participated voluntarily and were tested individually, face-to-face. In return for their contribution, they received a small monetary reward. All of them were native German speakers and had normal or corrected-to-normal vision. The study was conducted in accordance with the Declaration of Helsinki and approved by the local Ethics Committee of the Medical School, University of Tübingen, Germany. Informed written consent was obtained from all participants, and the data were processed anonymously.

### Body language reading: point-light locomotion in a BME task

**Fig. 1:**
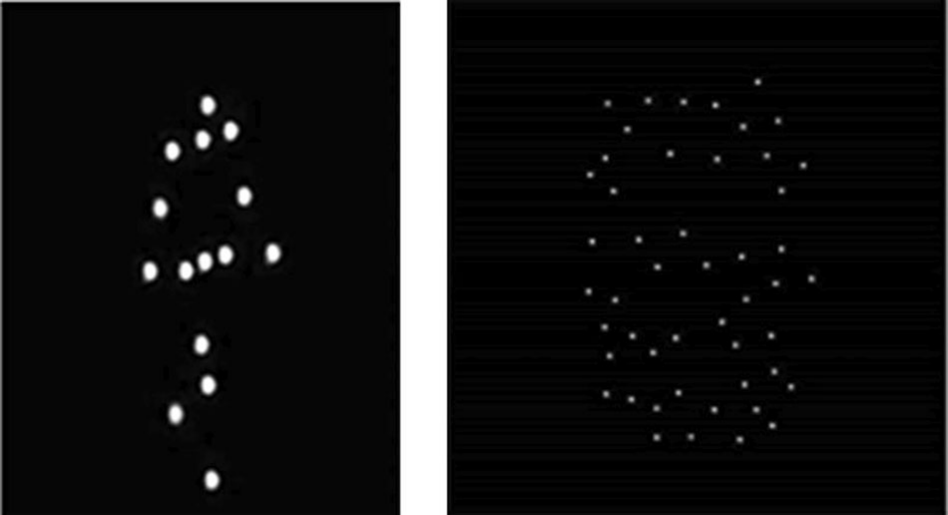
Example of stimuli used. Left, a static frame from a dynamic sequence portraying a walking person facing right in an intermediate position between the frontal and sagittal views. Right, a frame from a dynamic sequence representing a point-light face of a female actor expressing anger. From Pavlova et al.^45^; the Creative Commons Attribution [CC BY] license.

The stimuli and the emotion through body/biological motion (BME) task are described in detail elsewhere^45^. In brief, a set of point-light black-and-white animations portraying human locomotion was presented to participants (Fig. 1). They were created by using the Motion Capture Library (N Stage, Pinewood Studios, Iver Heath, Buckinghamshire, United Kingdom)^43,76,77^. Each point-light display was built of 15 white dots that were placed on the shoulder, elbow, and wrist on each arm; on the hip, knee, and ankle of each leg; and on the head, neck, and pelvis of an otherwise invisible body. The pelvis of the walker was fixed to the middle of the screen. Each movie was shown for 2 s that corresponded to one walking cycle consisting of two steps. During locomotion, the walker was seen facing right in the intermediate position of 45° between the frontal and sagittal views. This intermediate position was used since the sagittal view is often considered neutral in social interactions, whereas the frontal view triggers ambiguous (facing either toward or backward an observer) and often gender-dependent impressions of locomotion direction (for details, see ref. ^45^). For creation of the left-facing walkers, the videos were rotated to 90° horizontally. To avoid variability in emotion portrayal, movies expressing different emotions (in this case, anger and neutrality) were produced with the same actor. The stimulus set was the same as in a previous study^45^. Each experimental session consisted of a set of 144 trials (4 actors [2 male/2 female] × 2 emotions [angry/neutral] × 2 facing directions [left/right] × 9 [3 repetitions of each stimulus × 3 runs]) presented in a pseudorandomized order. In a two-alternative forced choice (2AFC) paradigm, participants were asked to indicate (by pressing a respective key) immediately after stimulus offset whether the locomotion expressed anger or neutrality. If participants failed to respond during the inter-stimulus interval, ISI (after stimulus offset till onset of the next stimulus right after the participant’s response) that randomly varied between 3 and 5 s, the next trial started automatically. On this task, 1.54 ± 2.29 responses were missed in the SZ group (Mdn, 1.00, 95% CI [0.65; 2.42]), while 0.21 ± 0.57 responses were missed in the TD group (Mdn, 0.00, 95% CI [−0.01; 0.44]).

### Face language reading: Point-light faces in the FME task

The dynamic point-light face displays were previously used in our lab for testing TD individuals^45^. Display production is described in detail elsewhere^78^. The stimuli were kindly shared with us by Dr. Anthony Atkinson. In brief, 50 small white dots were positioned in a quasi-random order on an actor’s face (Fig. 1). To ensure an even distribution of the dots, the face was divided into four quadrants, with the tip of the nose as a center, where two imaginary lines, horizontal and vertical, met. Each quadrant contained approximately the same number of white dots. The quasi-random placement minimized the availability of structural information, such as from areas of the lips, cheeks, or eyebrows. No dots were placed on the eyelids. Still, some static form cues could not be prevented, such as dark regions at the position of the eyes and the opening of the mouth. The displays had been proven for recognizability in earlier behavioral and neuroimaging studies^45,78^. The movies of 6 (3 female/3 male) actors with happy and angry expressions were presented in pseudorandomized order in 3 separate runs with a short break between them. In total, each experimental session consisted of 108 trials (6 actors [3 female/3 male] × 2 emotions [happy/angry] × 3 displays for each emotion by each actor × 3 repetitions of each stimulus). In a 2AFC paradigm, participants had to indicate (by pressing one of two respective keys) facial affect. Each video lasted 2 s. Participants were asked to respond right after stimulus offset. During an ISI that was randomly jittered between 3 and 5 s, a white fixation cross was displayed in the center of the screen. If participants failed to respond within this period, the next trial automatically started. On the emotion through face motion (FME) task, 1.61 ± 3.58 responses were missed in the SZ group (Mdn, 0.00; 95% CI [0.22; 3.00]), while 0.11 ± 0.32 responses were missed in the TD group (Mdn, 0.00; 95% CI [−0.01; 0.24]).

Both the BME and FME tasks were administered to participants as a computer version by using Presentation software (Neurobehavioural Systems, Inc., Albany, CA, USA). In each task, recognition accuracy was calculated as the ratio of the number of trials with correct responses to the total number of trials, in which this correct response was expected. Response time (RT) was calculated for correct trials from the stimulus offset to the response. Instructions were carefully explained, and each participant was administered a short pre-test to control for instruction understanding before the examination began. No immediate feedback regarding performance was given to participants. The session with the BME and FME tasks lasted for about 30–35 min.

### Data analysis

All data sets were routinely checked for normality of distribution with the Shapiro–Wilk test, followed by the use of either parametric (for normally distributed data) or non-parametric statistics. For non-normally distributed data, in addition to means and SDs, Mdn and 95% CIs are reported. Inferential statistics were performed by mixed-model analyses of variance, ANOVA, and post-hoc pairwise comparisons using JMP software package (version 16.2, SAS Institute, Cary, North Carolina, USA). Non-parametric statistics (Mann-Whitney test and Wilcoxon signed-rank test) were performed for between-group and within-group comparisons, respectively, using MATLAB (version 2023a; MathWorks Inc., Natick, MA, USA). For within-group correlation analyses, Pearson product-moment correlation was used for normally distributed data, whereas Spearman’s rank correlation was applied to non-normally distributed data. One-tailed tests were used when effects were expected on the basis of previous findings and reports, while two-tailed tests were implemented in the absence of such expectations. False discovery rate (FDR) corrections for multiple comparisons had been applied separately for accuracy and RT analyses. Uncorrected *p*-values are reported (only if different from corrected), as corrected values can lead to an increase in false negatives.

## Results

### Recognition accuracy

**Fig. 2:**
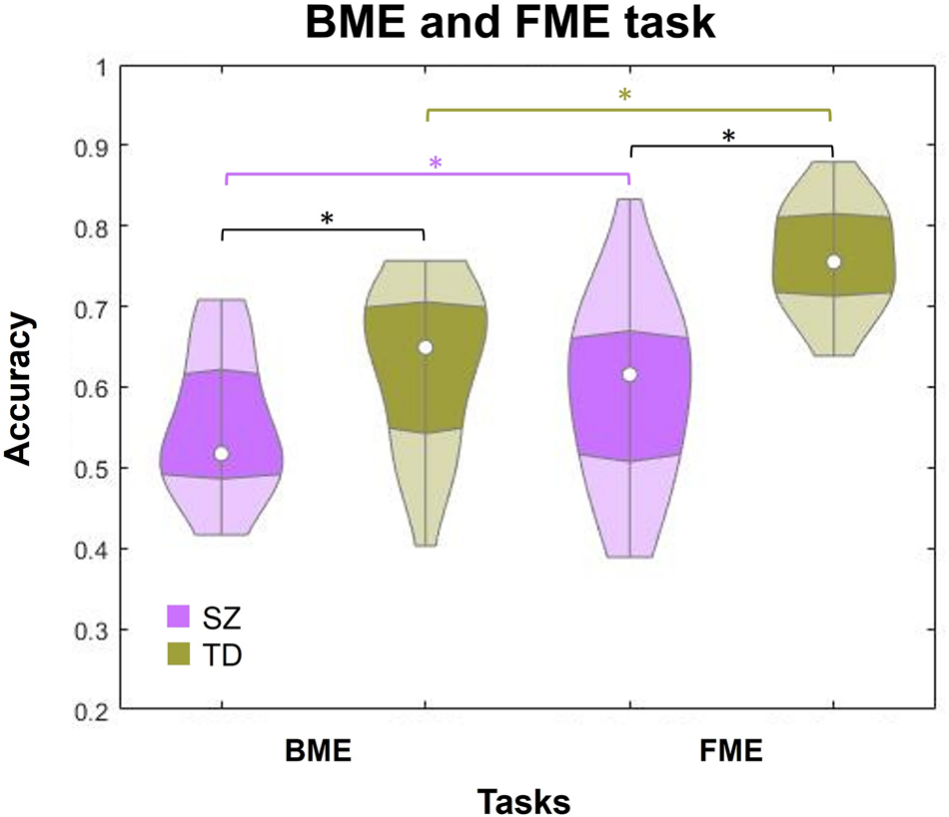
Violin plots of accuracy on the BME and FME tasks in SZ (purple, left violins in each pair) and TD (olive green, right violins in each pair) males. Asterisks indicate significant between- and within-group differences (*p* < 0.05).

The individual accuracy rates were submitted to a two-way mixed-model ANOVA with the within-subject factor Task (BME/FME) and between-subject factor Disorder (Yes/No). A main effect of Task was highly significant (*F*(1,54) = 46.20, *p* < 0.001; effect size, *partial eta-squared η*^*2*^_*p*_ = 0.46), with better performance on the FME task. A main effect of Disorder was also highly significant (*F*(1,54) = 88.36, *p* < 0.001; *η*^*2*^_*p*_ = 0.62), with TD individuals outperforming SZ patients. A Disorder by Task interaction was significant (*F*(1,54) = 12.69, *p* < 0.001; *η*^*2*^_*p*_ = 0.19). On both tasks, emotion recognition accuracy was lower in SZ patients than in their TD peers (BME, 0.551 ± 0.087 and 0.627 ± 0.097, for SZ and TD, respectively; *t*(54) = 3.07, *p* = 0.004 [uncorrected, *p*_*uncorr*_ = 0.003]; here and further two-tailed (if not stated otherwise) and false discovery rate [FDR] corrected for multiple comparisons, uncorrected *p*-values are provided only if different from corrected; effect size, Cohen’s *d* = 0.82; FME, 0.593 ± 0.109 and 0.761 ± 0.062, for SZ and TD, respectively; *t*(54) = 7.04, *p* < 0.001; *d* = 1.88; Fig. 2). Both groups showed higher emotion recognition accuracy on the FME task compared to BME task (for SZ, *t*(27) = 2.36; *p* = 0.026; *d* = 0.42; for TD, *t*(27) = 7.12, *p* < 0.001; *d* = 1.62).

**Fig. 3:**
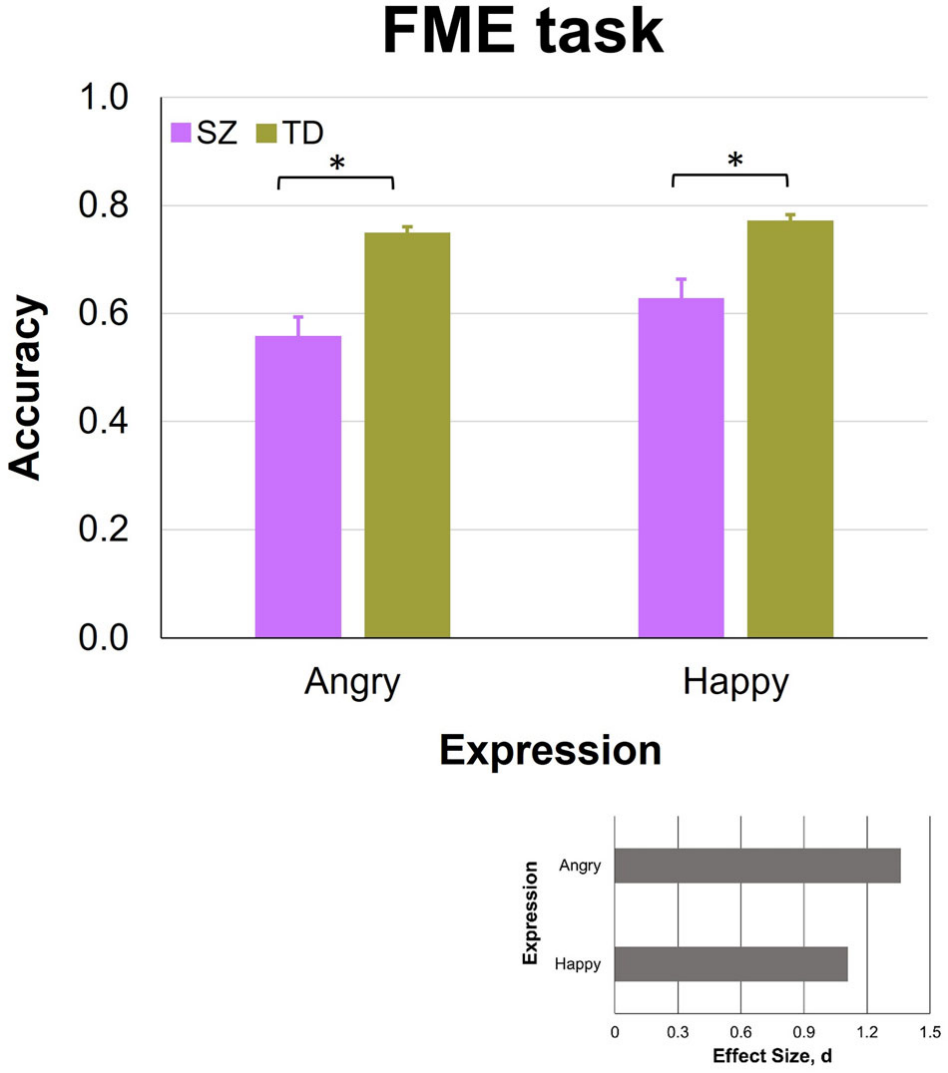
Accuracy rates for angry and happy expressions on the FME task in SZ (purple, left bars in each pair) and TD (olive green, right bars in each pair) males. Vertical bars represent ±SEM. Asterisks indicate significant differences (*p* < 0.05). Effect sizes for comparisons between TD and SZ participants are shown in the inset (right bottom plot).

To gain a more detailed insight into emotion recognition in SZ, we compared accuracy for each emotion (angry and happy) separately on the FME task. With this purpose in mind, the individual accuracy rates for angry and happy facial expressions were submitted to a two-way mixed-model ANOVA with the within-subject factor Expression (Angry/Happy) and between-subject factor Disorder (Yes/No). A main effect of Disorder was highly significant (*F*(1,54) = 38.31, *p* < 0.001; *η*^*2*^_*p*_ = 0.42), with SZ patients underperforming on the task. A main effect of Expression (*F*(1,54) = 2.90, *p* = 0.090, n.s.; *η*^*2*^_*p*_ = 0.05) and a Disorder by Expression interaction were not significant (*F*(1,54) = 0.80, *p* = 0.376, n.s.; *η*^*2*^_*p*_ = 0.02). The lack of interaction indicates that for both emotional expressions, TD individuals were to a similar degree more accurate than SZ patients (Fig. 3). Pair-wise analysis also revealed that compared to their TD peers, SZ patients exhibited lower recognition accuracy for both facial emotions: anger (for SZ, 0.558 ± 0.185; for TD, 0.750 ± 0.072; *t*(54) = 5.10, *p* < 0.001; *d* = 1.36) and happiness (for SZ, 0.628 ± 0.151; for TD, 0.772 ± 0.102; *t*(54) = 4.16, *p* < 0.001; *d* = 1.11). No difference in recognition accuracy between angry and happy point-light faces was found within the TD group (*t*(27) = 0.93, *p* = 0.360, n.s.; *d* = 0.25) as well as in SZ (*t*(27) = 1.44; *p* = 0.215 [*p*_*uncorr*_ = 0.161], n.s.; *d* = 0.35).

The BME task has proven to be rather difficult not only for SZ patients, but also for TD controls, mainly because of the low general recognizability of displays representing angry walking. In our opinion, the reason for the lower recognizability lies in the less pronounced expressions of anger displayed by the actors. Both SZ and TD individuals were better at recognizing neutral than angry walking (SZ, *t*(27) = 2.69, *p* = 0.024 [*p*_*uncorr*_ = 0.012]; *d* = 0.45; TD, *t*(27) = 4.68, *p* < 0.001; *d* = 1.07). There was no difference in recognition accuracy of angry walkers between SZ and TD individuals (for SZ, 0.461 ± 0.200; for TD, 0.515 ± 0.144; *t*(54) = 1.17, *p* = 0.250, two-tailed; n.s.; *d* = 0.31). As expected from earlier work^29,71^, the recognition of neutral body motion was more accurate in the TD than SZ group (for SZ, 0.642 ± 0.196; for TD, 0.739 ± 0.173; *t*(54) = 1.96, *p* = 0.038 [*p*_*uncorr*_ = 0.028], one-tailed; *d* = 0.52).

### Link in recognition accuracy between BME and FME

**Fig. 4:**
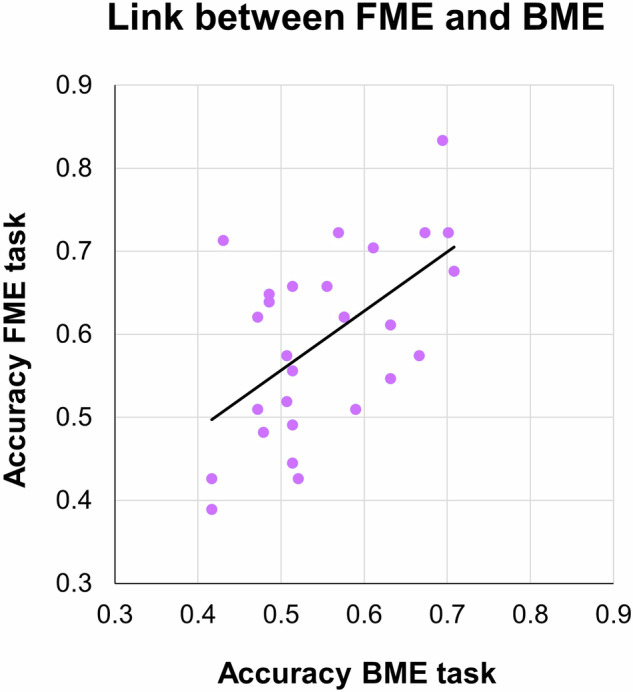
Link between recognition accuracy on the FME and BME tasks in SZ patients as indicated by a significant positive linear Pearson correlation (*p* = 0.002).

As reading point-light dynamic faces and bodies may rely upon similar dis/abilities, we expected to find a link in recognition accuracy between these two tasks. As expected, a positive correlation between recognition accuracy on the BME and FME tasks was found in SZ patients (Pearson product-moment correlation, *r*(27) = 0.563, *p* = 0.002; Fig. 4). However, it was absent in TD controls (*r*(27) = 0.279, *p* = 0.150, n.s.). The lack of a link between BME and FME recognition accuracy in TD individuals agrees with previous findings obtained in an independent sample of young TD males aged 19–31^45^.

### Response time

The individual response time (RT) values for correct responses on the BME and FME tasks were submitted to a two-way mixed model ANOVA with the between-subject factor Disorder (Yes/No) and within-subject factor Task (BME/FME). A main effect of Disorder was highly significant (*F*(1,54) = 43.92, *p* < 0.001; effect size, *partial eta-squared η*^*2*^_*p*_ = 0.45), with SZ patients responding slower than their TD peers. A main effect of Task (*F*(1,54) = 0.08, *p* = 0.777, n.s.; *η*^*2*^_*p*_ = 0.002) as well as a Disorder by Task interaction (*F*(1,54) = 0.11, *p* = 0.737, n.s.; *η*^*2*^_*p*_ = 0.002) were not significant.

As expected from earlier work on nonverbal social cognition in SZ^4,17^, the main outcome indicates that TD controls were faster than SZ patients in providing correct responses on the BME task (SZ, 0.796 ± 0.324 s; TD, 0.603 ± 0.197 s; *t*(54) = 2.69, *p* = 0.007 [*p*_*uncorr*_ = 0.005], one-tailed; here and further FDR corrected; effect size, Cohen’s *d* = 0.72) as well as on the FME task (SZ, 0.794 ± 0.366 s; Mdn, 0.720, 95% CI [0.652; 0.936]; TD, 0.620 ± 0.190 s; *U* = 280, *p* = 0.034, one-tailed; effect size, *r* = 0.24). A positive correlation was found for processing speed (RT) on the BME and FME tasks in both SZ (Spearman’s *rho*, *ρ*(27) = 0.785, *p* < 0.001) and TD individuals (Pearson product-moment correlation*, r*(27) = 0.663, *p* < 0.001; Fig. S1, Supplementary Material). The link between BME and FME processing speed (RT) in TD individuals is in line with previous findings obtained in an independent sample of young TD males aged 19–31^45^.

## Discussion

The objective of the present study was to investigate the ability to infer emotions from dynamic point-light displays of faces and bodies in male SZ. The findings demonstrate that: (i) Patients with SZ exhibit a general impairment in inferring emotions from the challenging tasks with point-light displays representing dynamic faces and bodies. (ii) In patients with SZ only, the accuracy of emotion recognition from point-light bodies and faces is interrelated, whereas both patients with SZ and their TD peers show a positive correlation between processing speed (in terms of RT) on these two tasks.

### Recognition of emotions through point-light body motion

The outcome is consistent with those of previous research, indicating that individuals with SZ underperform on a variety of point-light body motion tasks. Patients demonstrate difficulties in discrimination of facing (right or left) of a point-light walker embedded into a simultaneous noise^79,80^ and in judging whether point-light displays resemble human movement^81^. They poorly recognize communicative cues in dyadic point-light displays (indicating whether one in a pair of point-light agents either imitates or follows actions performed by the other one^33,34^), and are poor in differentiation between communicative/interactive and independent actions of two agents^28,31,32^ as well as of a single point-light agent^34^. Individuals with SZ often misperceive non-communicative actions as communicative ones^28^. Moreover, SZ patients are less accurate in discrimination of point-light human displays from similar scrambled configurations, and the sensitivity (as assessed by the signal-detection index *d*-prime) to point-light body motion is positively linked to impaired social functioning as measured by the Zigler social competence scale^82^. Moreover, patients with SZ show a positive correlation between detection of a camouflaged walker and affective empathy index^83^.

The present findings dovetail with earlier work in the sense that they indicate substantial deficits in inferring social signals from point-light body motion in SZ^29,30,34,72,84,85^. As indicated by a meta-analysis^35^, patients with SZ typically underperform on revealing emotions from body motion tasks with an effect size of 0.61. Notably, most of the studies are based on videotapes of only one (either female or male) or two (female and male) actors. The vast majority of studies on emotion recognition from body motion in psychiatric populations use videos of only one male actor, such as the *EmoBio* test first introduced by Heberlein with colleagues^86^. This could potentially limit the generalization of the findings.

Individuals with SZ who display increased violence risk, in particular, committed homicide, also exhibit general deficits in emotion recognition through body motion and a tendency to undermentalize, i.e., to fall short of ascribing mental states and emotions to others^75,85^. Patients with SZ (predominantly males; 85 participants, 54 males) are less accurate than TD controls in emotion recognition (anger, happiness, sadness, fear, and neutral expressions)^29^. The recognition accuracy of emotions from body motion shows a comparable profile in both SZ individuals and healthy controls, exhibiting similar fluctuations, namely, slightly higher accuracy in recognizing happiness and neutral expressions compared to other (negative) emotions.

However, later work^75^ indicates that non-violent patients with SZ (males only) experience more difficulties in recognition of some emotions as compared to others: in other words, the mean recognition accuracy of SZ patients for some emotions (in SD units) deviates from (is lower than) the mean accuracy of TD controls more than for the others. SZ patients do not differ from controls in recognition of anger and sadness, but experience more difficulties in recognizing fearful locomotion^75^. The controversy in reporting either global or selective deficits in emotion recognition in SZ may be attributed to the inhomogeneity of samples. In our view, patients with severe general cognitive impairments are more likely to exhibit global deficits in inferring emotions, whereas individuals with mild impairments and less pronounced SZ symptoms are more likely to have selective deficits. In a nutshell, this assumption agrees well with the study of social cognitive heterogeneity in individuals with SZ, which indicates that social cognitive tasks can be used for clinical and cognitive subtyping with substantial differences between sub-groups in functioning, symptom load, and nonsocial (*cold*) cognition^36^. An additional account for the controversy surrounding global and selective deficits is that a global deficit arises when either visual input (social signals) is limited or the task is too demanding.

### Recognition of emotions through point-light face motion

Individuals with SZ often demonstrate deficient facial affect recognition, which is considered a core feature of nonverbal daily social cognition^27,28^. Symptom severity in SZ negatively correlates with the facial expression recognition ability^87^. As these deficits are more pronounced in patients with chronic SZ, it is likely that they increase with duration of the disorder^88^.

The present findings reveal that inferring emotions in point-light dynamic faces is impaired in SZ. Most importantly, the ability to read point-light faces is globally impaired, with both displayed facial affects (anger and happiness) being recognized less accurately. This outcome aligns with the only (to our knowledge) earlier study that investigated emotion recognition from point-light faces in individuals with SZ^89^. Inferring facial affect in SZ patients was reported to be proportionally less accurate for all emotions (anger, happiness, surprise, sadness, fear, and disgust). The recognition profile was uneven for distinct emotions but rather similar in both SZ and TD individuals, with some (negative) emotions (such as disgust, anger, and fear) being slightly less accurately detectable than others, such as surprise or happiness. In general, both studies (the present one and ref. ^89^) point to the global deficit in inferring emotions from point-light dynamic faces.

### Global versus selective deficits

As pointed out earlier^17^ and above, no consensus has been reached so far in favor of selective (deficits only in distinct emotions or valence) versus global (for all emotions, either even or uneven in size) impairments in inferring social signals in SZ. Some work stands for a global deficit, particularly in recognition of *negative* emotions, and considers this impairment as a vulnerability indicator^90^, trait marker^91^, or heritable endophenotype^92^. Other researchers argue that impairments are rather selective, affecting primarily recognition of fear and anger^93^, or surprise, contempt, sadness, disgust, and neutral expressions (but not happiness, anger, and fear)^51^, or happiness and surprise^94^.

**Fig. 5:**
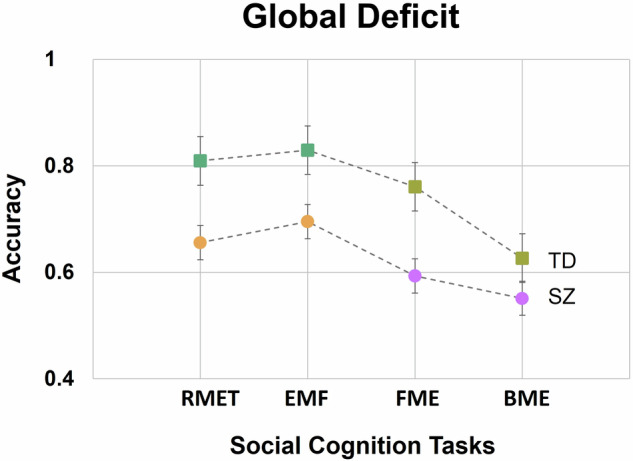
Comparison of accuracy rates in SZ and TD individuals for FME and BME tasks (the present work, coded as olive green for TD and purple for SZ) and the RMET and EMF task (coded as ocean wave for TD and apricot for SZ) from Resch et al.^17^; the Creative Commons Attribution [CC BY] license.

The present findings uncover global deficits in inferring emotions through dynamic body language as well as through dynamic faces. This outcome agrees well not only with previous research in independent cohorts of SZ patients^29,89^, but also with earlier work on reading emotions in the static eyes with the same cohort of individuals with SZ as in the present study^17^. As with dynamic faces and bodies, while the recognition pattern is uneven across different emotions/mental states in faces covered by masks (EMF) and in the modified Reading the Mind in the Eyes Test (RMET-M), it is similar in both SZ and TD individuals^17^. Reduction in performance across the social cognition tasks in the same sample of SZ patients is largely uniform (Fig. 5).

This raises the question of the origins of a global impairment in emotion recognition in SZ that requires further research: does a proportional decrease in emotion recognition accuracy reflect the specificity of nonverbal social cognition or rather stem from other nonsocial cognitive deficits, symptom load, and everyday functioning that constitutes the very nature of SZ?

### Relationship between inferring emotions in dynamic faces and bodies

Social signals from dynamic faces and bodies are of vital value for effective interaction. In accord with this, a recent meta-analysis^95^ reveals that body posture has as great an impact on emotion recognition as facial expressions. Signal clarity and emotional congruency determine whether individuals rely more heavily on facial or bodily cues^95^. Given that social signals from both faces and bodies are essential for adaptive social cognition and interaction, we examined whether inferring emotional signals from point-light faces is related to emotion recognition through dynamic bodies in SZ and TD individuals. The outcome demonstrates that a positive correlation between the accuracy of reading dynamic faces and bodies occurs in SZ, albeit not in TD individuals. The lack of this link in male TD individuals is consistent with previous findings obtained in an independent cohort of young TD males aged 19–31^45^ and may reflect the specificity of perceptual strategies in reading point-light faces and bodies: individuals who are successful in reading dynamic faces may be less accurate in body reading, and vice versa. One potential explanation for the intimate link between reading the language of faces and bodies in SZ is that the disease (and its severity) affects both abilities.

As the outcome of this study shows, processing speed (as reflected in RT) of inferring social signals through dynamic bodies and faces is closely tied both in SZ and TD individuals (Fig. S1 in Supplementary Materials). As pointed out earlier^17^, this suggests a commonality in encoding of the social signals and accumulation of sensory evidence. In accord with drift-diffusion models of decision making^96,97^, *non-decision* processing time (time needed to process sensory information and to execute a motor response) is crucial for individual RT variability. On the same wavelength, as already mentioned above, a lack of association between the accuracy of body and face reading suggests *distinct* latent neurocognitive mechanisms underpinning reading faces and bodies, especially accumulation of sensory evidence for decision making, as may be the case with TD males in the present study. By contrast with their TD peers, for SZ patients, inferring emotions from dynamic faces and bodies may be rather challenging in terms of neurocognitive mechanisms and decision making, which is reflected in the tight link in recognition accuracy.

To conclude, this work offers novel insights into the global deficits in reading the language of dynamic bodies and faces in SZ and provides a blueprint for the development of strategies to target these impairments. More generally, the study sheds light on the profile of social cognition deficits in mental disorders that differ in their gender prevalence, manifestation, severity of symptoms, and other disease characteristics. To gain a better understanding of social cognition in SZ, further research in female patients is required to ascertain whether these deficit patterns generalize across genders.

## Supplementary information

Supplementary Material

## Data Availability

Data is provided within the paper or supplementary information files.
