# Supplementary material for: Reading body and face language in male schizophrenia
Source: Schizophrenia (Heidelb). 2026 Jul 7;12(1):61. doi: 10.1038/s41537-026-00775-6 (PMC13346531; doi:10.1038/s41537-026-00775-6)
Supplement: Supplementary file 1 — Supplementary Material [file 41537_2026_775_MOESM1_ESM.pdf]

SUPPLEMENTARY MATERIAL

Reading body and face language in male schizophrenia

Annika Resch[1], Alexander N. Sokolov[1], Patrick Steinwand[1], Andreas J. Fallgatter[1,2], Marina A. Pavlova[1,*]

[1] Department of Psychiatry and Psychotherapy, Tübingen Center for Mental Health (TüCMH), Medical School and University Hospital, Eberhard Karls University of Tübingen, Tübingen, Germany

[2] German Center for Mental Health (DZPG), partner site Tübingen, Germany

* Correspondence: Prof. Marina A. Pavlova, Calwerstr. 14, 72076 Tübingen, Germany; tel: +49 7071 2981419; E-mail: marina.pavlova@uni-tuebingen.de

SUPPLEMENTARY METHODS

Patient information

Twenty-seven out of 28 SZ patients were under medication. Most of them received atypical antipsychotics such as olanzapin (13), aripiprazol (6), risperidon (5), clozapin (4), quetiapin (3), amisulprid (2), sulpirid (1); and/or typical antipsychotics such as haloperidol (3), perazin (1), pipamperon (2); and/or antidepressants [mirazapin (3), sertralin (2), escitalopram (2), venlafaxin (2), citalopram (1), duloxetin (1); lithium (4)]; benzodiazepines such as lorazepam (5); and anticonvulsants such as carbamazepin (1).

Table S1. Comorbidity in SZ patients

	**Comorbidities (ICD-10 Code)**
P01	Postschizophrenic depression (F20.4)<br>Mental and behavioral disorders due to use of tobacco, dependence syndrome (F17.2)
P02	Mental and behavioral disorders due to use of cannabis, dependence syndrome (F12.2)<br>Mental and behavioral disorders due to use of tobacco, dependence syndrome (F17.2)<br>Mental and behavioral disorders due to use of alcohol, harmful use (F10.1)<br>Mental and behavioral disorders due to use of cocaine, harmful use (F14.1)<br>Mental and behavioral disorders due to use of other stimulants, including caffeine, harmful use (F15.1)
P04	Mental and behavioral disorders due to use of tobacco, dependence syndrome (F17.2)
P05	Mental and behavioral disorders due to use of tobacco, dependence syndrome (F17.2)
P06	PTSD/post-traumatic stress disorder (F43.1)
P07	Mixed and other personality disorders (F61)<br>Conduct disorder (F91)<br>Mental and behavioral disorders due to use of tobacco, dependence syndrome (F17.2)
P08	Mental and behavioral disorders due to use of tobacco, dependence syndrome (F17.2)<br>Mental and behavioral disorders due to the use of cannabis, dependence syndrome (F12.2)<br>Mental and behavioral disorders due to use of other stimulants, including caffeine, harmful use (F15.1)
P09	Obsessive compulsive disorder (F42.1)<br>Mild mental retardation (F70.0)
P11	Pervasive developmental disorder (F84.9)<br>Mixed and other personality disorders (F61)
P14	Mental and behavioral disorders due to use of alcohol, dependence syndrome (F10.2)
P15	Mental and behavioral disorders due to use of tobacco, dependence syndrome (F17.2)
P16	Mental and behavioral disorders due to use of tobacco, dependence syndrome (F17.2)
P19	Mental and behavioral disorders due to use of tobacco, dependence syndrome (F17.2)
P20	Recurrent depressive disorder (F33.2)<br>Disturbance of activity and attention (F90.0)
P24	Mental and behavioral disorders due to use of other psychoactive substances, harmful use (F19.1)
P26	Mental and behavioral disorders due to use of cannabis, dependence syndrome (F12.2)<br>Mental and behavioral disorders due to use of other psychoactive substances, harmful use (F19.1)
P27	Mental and behavioral disorders due to use of opioids, dependence syndrome (F11.2)<br>Mental and behavioral disorders due to use of cannabis, dependence syndrome(F12.2)<br>Mental and behavioral disorders due to use of tobacco, dependence syndrome (F17.2)

SUPPLEMENTARY RESULTS

Link in processing speed between BME and FME task

A positive correlation occurred between RT on the BME and FME task for both patients with SZ ($\rho(27) = 0.785$, $p < 0.001$) and TD individuals ($r(27) = 0.663$, $p < 0.001$; **Fig. S1**).

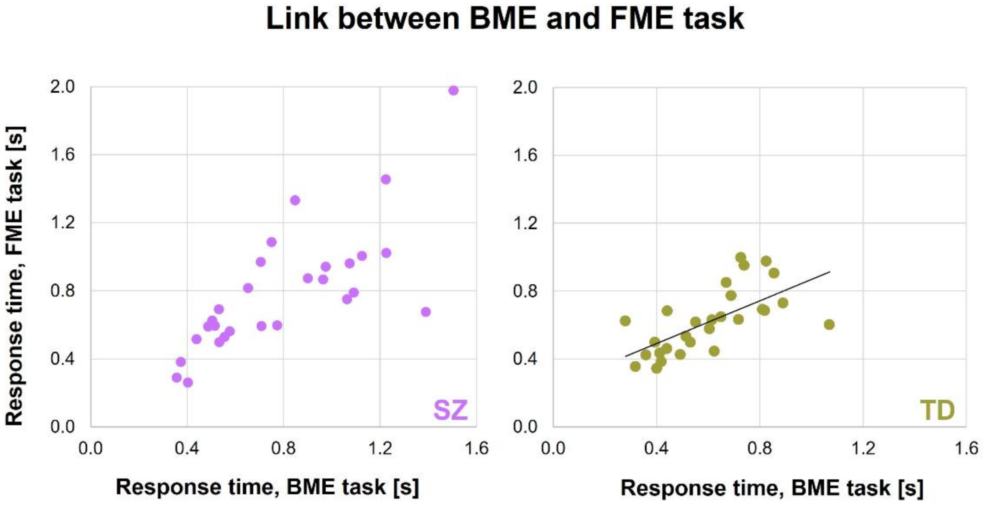

Figure S1. Link between response time (RT) on the FME and BME tasks in SZ patients (lila, Spearman's *rho*, $\rho(27) = 0.785$, $p < 0.001$) and TD controls (olive green) as indicated by significant positive linear Pearson correlation ($r(27) = 0.663$, $p < 0.001$).

Link in recognition accuracy on BME and FME tasks and age

In SZ, no link was found between chronological age and accuracy on the BME task (SZ, $\rho(27) = -0.212$, $p = 0.278$; n.s.) or age and accuracy on the FME task (SZ, $\rho(27) = -0.011$, $p = 0.955$; n.s.). In TD controls, a correlation between age and accuracy only tended to be significant on the BME task ($\rho(27) = -0.354$, $p = 0.065$; n.s.) and did not occur on the FME task ($\rho(27) = -0.238$, $p = 0.222$; n.s.).
